# Building communication networks: international network for the study and prevention of emerging antimicrobial resistance.

**DOI:** 10.3201/eid0702.010235

**Published:** 2001

**Authors:** H M Richet, J Mohammed, L C McDonald, W R Jarvis

**Affiliations:** Centers for Disease Control and Prevention, Atlanta, Georgia, USA. herve.richet@chu-nantes.fr

## Abstract

The global nature of antimicrobial resistance and the failure to control the emergence of resistant organisms demand the implementation of a global surveillance program involving both developed and developing countries. Because of the urgent need for infection control interventions and for rapid distribution of information about emerging organisms, we initiated the International Network for the Study and Prevention of Emerging Antimicrobial Resistance (INSPEAR). Its main objectives are to serve as an early warning system for emerging antimicrobial-drug resistant pathogens, to facilitate rapid distribution of information about emerging multidrug-resistant pathogens to hospitals and public health authorities worldwide, and to serve as a model for the development and implementation of infection control interventions.


### Building Communication Networks: International Network for the Study and Prevention of Emerging Antimicrobial Resistance

**Hervé M. Richet,* Jasmine Mohammed,* L. Clifford McDonald,† William R. Jarvis,* and INSPEAR[1]**
*Centers for Disease Control and Prevention, Atlanta, Georgia, USA;
†National Health Research Institute, Taipei, Taiwan

The global nature of antimicrobial resistance and the failure to control the emergence of resistant organisms demand the implementation of a global surveillance program involving both developed and developing countries. Because of the urgent need for infection control interventions and for rapid distribution of information about emerging organisms, we initiated the International Network for the Study and Prevention of Emerging Antimicrobial Resistance (INSPEAR). Its main objectives are to serve as an early warning system for emerging antimicrobial-drug resistant pathogens, to facilitate rapid distribution of information about emerging multidrug-resistant pathogens to hospitals and public health authorities worldwide, and to serve as a model for the development and implementation of infection control interventions.

The emergence of resistance to antimicrobial agents is becoming a major public health problem worldwide, especially in hospital-acquired infections. Infectious diseases experts are particularly concerned because organisms resistant to available antimicrobial drugs have been isolated in hospitals worldwide. The extent of antimicrobial-drug resistance in developing countries, where inappropriate antimicrobial usage may be more common, is unknown (1-6). The emergence and spread of these multidrug-resistant pathogens demonstrate that the medical community (including laboratories) may have difficulty isolating and identifying these organisms and that infection control interventions are either not implemented, ineffective, or implemented so late that the organism(s) has become endemic; in these circumstances, infection control strategies are not effective, and transmission continues (7,8).

The emergence of infections caused by methicillin-resistant *Staphylococcus aureus* (MRSA) vividly demonstrates this failure in infection control. MRSA emerged in Europe nearly 35 years ago concomitantly with the introduction of methicillin; subsequently, during the mid-1980s, epidemic strains spread in hospitals throughout the world. In many hospitals, few, if any, infection control precautions were implemented until recently, by which time these strains had become endemic, with infection rates approaching one per 100 admissions (9-12). The large reservoir of MRSA-colonized or -infected patients at these hospitals complicates infection control interventions.

Epidemiologic studies on antimicrobial resistance have alerted the medical and public health communities about the importance of emergence of antimicrobial resistance. However, most data on antimicrobial-resistant pathogens were collected as part of studies sponsored by the pharmaceutical industry, and most of the studies were methodologically flawed; thus, the data were not useful for generalizations about antimicrobial resistance in hospitals. In addition, in many countries, a close interrelationship does not exist between the laboratory identifying multidrug-resistant pathogens and the infection control personnel responsible for the prevention and control of transmission of such isolates. Furthermore, many laboratory-based surveillance systems are designed for making patient treatment decisions; the data are not organized in a way that can be used to design and implement control and prevention interventions.

With the development of the Emerging Infections Plan at the Centers for Disease Control and Prevention (CDC) and the endorsement and adaptation of this plan by the World Health Organization (WHO), the emergence of antimicrobial resistance has become a public health priority (13,14). Public health authorities in the United States and Europe realize that the emergence of antimicrobial-drug resistance is a global problem; no country is spared, and resistant organisms emerging in one country are likely to spread to other countries. With increasing travel and patient movement throughout the world, the situation exists for transmission of multidrug-resistant pathogens from one country or continent to another (15-19).

Because of the urgent need for infection control interventions to prevent further emergence of antimicrobial drug-resistant strains and for a rapid distribution of information about emerging organisms, we initiated the International Network for the Study and Prevention of Emerging Antimicrobial Resistance (INSPEAR). The main objectives of INSPEAR are to serve as an early warning system for emerging resistant pathogens, to facilitate rapid distribution of information about emerging multidrug-resistant pathogens to hospitals and public health authorities

Address for correspondence: Hervé M. Richet, Institut de Biologie des Hôpitaux de Nantes, CHU de Nantes, 44093 Nantes cedex 01, France; fax: (33) 2 40 08 38 29; e-mail: herve.richet@chu-nantes.fr

[1]D. Pittet, D.F. Brown, A.G. Dean, S. Gordon, I.M. Gould, M. Gubina, P. Heczko, M. Pana, J. Schindler, M.J. Struelens, F. Tenover, J. Vuopio-Varkila, A. Widmer, W. Witte.

worldwide, and to serve as a model for the development and implementation of infection control interventions to prevent the emergence or transmission of antimicrobial drug-resistant pathogens in health-care facilities. Another important function of INSPEAR is to assist microbiologists and infection control personnel in hospitals and countries that lack the expertise needed to conduct microbiologic or epidemiologic studies.

### Background

INSPEAR was begun as a collaborative effort between the Hospital Infections Program (CDC) and microbiologists and hospital epidemiologists in the United States and Europe. It is now a consortium of clinical microbiologists, hospital epidemiologists, infectious diseases specialists; experts in the fields of antimicrobial resistance, hospital epidemiology, and computer sciences; public health agencies; and national reference laboratories. One hundred sixty health-care facilities in 40 countries have joined INSPEAR, with 50% of participants in Western Europe and 29% in Eastern Europe.

### Recent Activities

Since its initiation in 1998, INSPEAR has conducted several activities essential to the implementation of the early warning system, such as the assessment of the way INSPEAR centers diagnose, conduct surveillance, and control infections caused by multidrug-resistant pathogens, as well as proficiency testing to ensure quality testing in laboratories participating in the program.

An assessment of MRSA infections, performed in 90 centers, was designed to assess the methods used by bacteriology laboratories to identify *S. aureus,* to determine the susceptibility of *S. aureus* to antimicrobial drugs, and to assess the surveillance and infection control programs in INSPEAR centers. This study revealed many deficiencies: Isolation of vancomycin- and teicoplanin-resistant *S. aureus* was reported by three centers but was not confirmed, and public health authorities were not alerted. Of the laboratories surveyed, 11% used oxacillin disks with antimicrobial content different from that recommended by the National Committee for Clinical Laboratory Standards or the Comité de L'Antibiogramme de la Société Française de Microbiologie; 20% did not have an internal quality control program; 36% did not participate in external quality control programs; and 14% did not determine MRSA susceptibility to vancomycin. Of the health-care facilities surveyed, 77% reported surveillance activities; however, only 36.5% determined the incidence rate per admission, and only 23% determined the rate per patient-days; 40% of the facilities did not have an MRSA control program; and 54% did not monitor or control the use of antimicrobial drugs. These data clearly demonstrate the urgent need to strengthen the laboratory and epidemiologic capacities of INSPEAR centers.

A proficiency testing study was performed to investigate the ability of INSPEAR centers to detect clinically important resistance phenotypes, to assist centers in establishing reliable methods to detect particular resistances, to validate data from hospital laboratories participating in INSPEAR, and to ensure consistent quality testing in INSPEAR clinical laboratories. Five strains were sent to the 116 participating laboratories: MRSA, hyper-beta-lactamase producing strain of *S. aureus*, glycopeptide-intermediate *Staphylococcus epidermidis*, and *van A* and *van B Enterococcus faecalis.* Seventy-six laboratories responded. Most laboratories did well with both *S. aureus* challenges; however, 60 (79%) had difficulty detecting reduced susceptibility to glycopeptides in staphylococci. All laboratories testing *van A E. faecalis* identified it correctly as vancomycin resistant, but the results for *van B E. faecalis* varied. Thirty-nine (52%) of 75 laboratories reported susceptible results for vancomycin, but 19% misidentified *van B E. faecalis* as vancomycin resistant. An assessment is being conducted to determine if participants have modified their testing methods based on the results of the proficiency testing and CDC recommendations.

### Early Warning System

Another reason antimicrobial resistance is uncontrolled is that clinical microbiology laboratories and the medical community often are not aware of emerging resistance and therefore are not prepared. Preparedness implies that potentially emerging events be known, that laboratorians have the capacity to detect emerging resistance and screen rapidly for colonization, that risk factors for emergence be assessed, and that health-care facilities have access to microbiologic and epidemiologic assistance and have the capacities for efficient isolation precautions (e.g., private rooms with handwashing facilities, availability of gloves). Therefore, to coordinate the timely international scientific and public health response to emerging antimicrobial resistance, we designed an early warning system to monitor, analyze, control, and prevent important events in the emergence of antimicrobial resistance at both the global and regional or local levels. Overall, this early warning system should trigger early epidemiologic and microbiologic interventions to assess risk factors for emerging antimicrobial resistance, leading to more effective control.

### Global Sentinel Events

The need to be aware of global sentinel events is leading to an important function of the program: the periodic publication of what INSPEAR members determine by consensus to be important types of antimicrobial resistance heretofore undescribed or of great public health importance (Table 1). Criteria used to arrive at this list included the overall ease of use of the list by most clinical and reference

Table 1. Early warning system: global sentinel events

| Microorganism | Resistance |
|---|---|
| *Streptococcus* spp. | Penicillinase, gentamicin, glycopeptides, fluoroquinolones |
| *S. pneumoniae* | Vancomycin, third-generation cephalosporins, new fluoroquinolones (gemifloxacine, grepafloxacin, levofloxacin, trovafloxacin) |
| *Staphylococcus* spp. | Glycopeptides (high level) |
| *Enterobacteriaceae* | Carbapenemase |
| *Neisseria meningitidis* | Penicillinase, chloramphenicol, cephalosporins, fluoroquinolones |
| *Acinetobacter baumannii* | Carbapenemase |
| *Salmonella typhi* | Third-generation cephalosporins, fluoroquinolones |
| *Haemophilus influenzae* | Cephalosporins |
| *Brucella* spp. | Tetracycline, rifampin, streptomycin |
| *Clostridium difficile* | Glycopeptides |
| *Clostridium perfringens* | Penicillinase |

laboratories and the actual and potential public health impact of resistance events based on factors such as pathogen virulence, frequency of infection caused by the pathogen, and absence of other licensed antimicrobial agents for treating infections caused by the pathogen. This list of events will be updated regularly and will be published and disseminated to national and international surveillance systems.

### Local and Regional Sentinel Events

Local and regional sentinel events consist of the first observation of a clinically important form of resistance in a particular locality or region. Such resistant phenotypes may already be well described from other localities or regions of the world (Table 2). The new regional emergence of resistance in an INSPEAR facility may warrant a coordinated response from local or international INSPEAR members to prevent the resistant strains from becoming endemic.

Table 2. Early warning system: local and regional sentinel events

| Microorganism | Resistance |
|---|---|
| *Staphylococcus aureus* | Methicillin, intermediate susceptibility to glycopeptides |
| *Enterococcus* spp. | Vancomycin |
| *Enterobacteriaceae* | Extended-spectrum beta-lactamase-mediated resistance, carbapenems, fluoroquinolones |
| *Acinetobacter baumannii* | Carbapenem |
| Any bacteria | All antimicrobials available at the regional and local settings |

### Functioning of the Early Warning System

The early warning system should function according to subsidiarity, defined as the principle that a central authority should have a subsidiary function, performing only tasks that cannot be performed effectively at a more immediate or local level. When an emerging event is suspected at an INSPEAR center, the national or regional coordinator should be alerted and microbiologic confirmation performed at the local, national, or regional level when possible, or with additional INSPEAR resources (Figure). Once an event is confirmed, the INSPEAR coordinator will be informed and the public health authorities, WHO, and the INSPEAR centers will be notified.

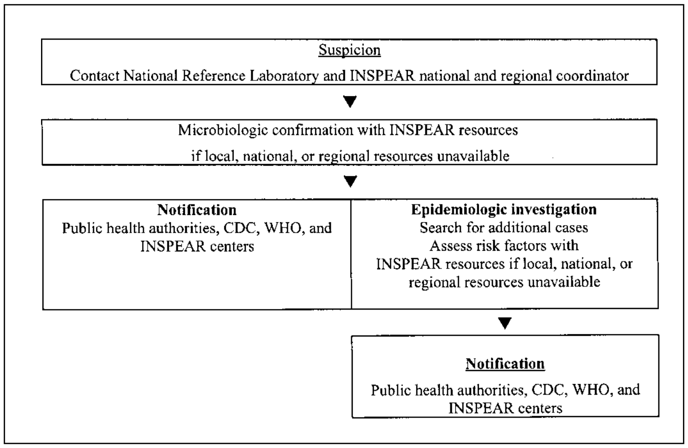

Figure. Functioning of the early warning system.

At the same time, an epidemiologic investigation will search for additional cases and assess risk factors through cohort or case-control studies, and surveillance will be implemented. As with microbiologic support, epidemiologic investigation will be performed at the local, national, or regional level if possible; if necessary, INSPEAR resources will be provided. In addition, public health authorities, WHO, and INSPEAR centers will be notified so that measures may be immediately implemented if such an event occurs elsewhere.

### Responses to Emerging Resistance

INSPEAR response may include immediate, specific responses, such as microbiologic support (e.g., confirmation of resistance, studying the mechanism of resistance, molecular typing to determine clonality) or on-site epidemiologic and infection control support (e.g., assistance with outbreak investigation, intervention studies, control measures) (Table 3). The level of response will be determined by local need, importance of the problem, and capacity of INSPEAR members to respond. In addition, the INSPEAR response to emerging resistance will include coordination of longer term studies to improve the methods for detection and control of resistance. Finally, the INSPEAR response will include the education and training of personnel at INSPEAR hospitals.

Table 3. INSPEAR resources

| Microbiology | Epidemiology |
|---|---|
| Bacterial identification | Surveillance system |
| Antimicrobial | |
| Resistance, testing, and characterization of mechanisms | Study design and conduct |
| Typing | Outbreak investigation |
| Quality control programs | Infection prevention interventions |
| Proficiency testing | Statistical analysis |
| Training | Statistical training |

### Conclusions

INSPEAR is the first international program dedicated to the control of antimicrobial resistance that combines microbiologic and epidemiologic expertise provided by national reference laboratories and public health agencies. This program should facilitate control of novel antimicrobial-resistant pathogens at the time of their emergence and increase the likelihood of controlling and preventing those pathogens before they become endemic. However, as the results of our MRSA survey and proficiency testing indicate, the microbiologic and epidemiologic capacities of health-care facilities worldwide will need to be strengthened if our goal of detection and control of multidrug-resistant pathogens is to be achieved.

Dr. Richet is head of hospital epidemiology and clinical bacteriology at the Centre Hospitalier Universitaire de Nantes (France) and professor of microbiology and hospital epidemiology at the Nantes School of Medicine. In 1987, he entered the Epidemic Intelligence Service at CDC in Atlanta and served as an EIS officer with the Hospital Infections Program and later as medical epidemiologist and returned to CDC from 1997 to 2000, where he served as a senior fellow, also with HIP.

### References

1. Go E, Urban C, Burns J, Kreiswirth B, Eisner W, Mariano N, et al. Clinical and molecular epidemiology of *Acinetobacter* spp. Infections sensitive only to polymixin and sulbactam. Lancet 1994;344:1329-32.
2. Richard P, Le Floch R, Chamoux C, Pannier M, Espaze E, Richet H. *Pseudomonas aeruginosa* outbreak in a burn unit: role of antimicrobials in the emergence of multiply resistant strains. J Infect Dis 1994;170:377-83.
3. French GL, Shannon KP, Simmons N. Hospital outbreak of *Klebsiella pneumoniae* resistant to broad-spectrum cephalosporins and beta-lactam-beta-lactamase inhibitor combinations by hyperproduction of SHV5 beta-lactamase. J Clin Microbiol 1996;34:358-63.
4. Neuwirth C, Siebor E, Pechinot A, Kazmierczak A. Outbreak of TEM-24-producing *Enterobacter aerogenes* in an intensive care unit and dissemination of the extended-spectrum beta-lactamase to other members of the family enterobacteriaceae. J Clin Microbiol 1996;34:76-9.
5. Morosini MI, Canton R, Martinez-Beltran J, Negri MC, Perez-Diaz JC, Baquero F,et al. New extended-spectrum TEM-type beta-lactamase from *Salmonella enterica* isolated in a nosocomial outbreak. Antimicrob Agents Chemother 1995;39:458-61.
6. Pagani L, Luzzaro F, Ronza P, Rossi A, Micheletti P, Porta F, et al. Outbreak of extended spectrum beta-lactamase producing *Serratia marcescens* in an intensive care unit. FEMS Immunol Med Microbiol 1994;10:39-46.
7. Johnson DR, Love-Dixon MA, Brown WJ, Levine DP, Downes FP, Hall WN. Delayed detection of an increase in resistant *Acinetobacter baumannii* at a Detroit hospital. Infect Control Hosp Epidemiol 1992;13:394-8.
8. Meyer KS, Urban C, Eagan JA, Berger BJ, Rahal JJ. Nosocomial outbreak of *Klebsiella* spp. Infection resistant to late-generation cephalosporins. Ann Intern Med 1993;119:353-8.
9. Barber M. Methicilin-resistant staphylococci. J Clin Pathol 1961;14:385-93.
10. Chabbert YA, Baudens JG. Souches de staphylocoques résistants naturellement à la méticilline et à la 5 méthyl-3-phényl-4-iso-oxazyl pénicilline (P12). Annales de l'Institut Pasteur (Paris) 1962;103:222-30.
11. Richet H, Wiesel M, Le Gallou F, Andre-Richet B, Espaze E. Methicillin-resistant *Staphylococcus aureus* control in hospitals: the French Experience. Infect Control Hosp Epidemiol 1996;17:509-11.
12. Pittet D, Safran E, Harbarth S, Borst F, Copin P, Rohner P, et al. Automatic alerts for methicillin-resistant *Staphylococcus aureus* surveillance and control: Role of a hospital information system. Infect Control Hosp Epidemiol 1996;17:496-502.
13. Baquero F, Task Force of the General Direction for Health Planning of the Spanish Ministry of Health. Antibiotic resistance in Spain: What can be done? Clin Infect Dis 1996;23:819-23.
14. Centers for Disease Control and Prevention. Addressing emerging infectious disease threats: a prevention strategy for the United States. Atlanta, GA: U.S. Department of Health and Human Services, Public Health Service, 1994.
15. Vandenbroucke-Grauls CMJE. Methicillin-resistant *Staphylococcus aureus* control in hospitals: The Dutch experience. Infect Control Hosp Epidemiol 1996;17:512-3.
16. Faber M, Rosdhal VT. Changing pattern of phage group II *Staphylococcus aureus* infection: from community to hospital. Scand J Infect Dis 1993;25:647-53.
17. Frenay HM, Van Leeuwen WJ, De Neeling AJ, Schot CS, Rost JA, Van Klingeren B. MRSA: Infection spreads between hospitals. BMJ 1994;308:58-9.
18. Harbarth S, Romand J, Frei R, Auckenthaler R, Pittet D. Transmission inter- et intrahospitaliere de staphylocoques dorés résistants à la méticilline. Schweiz Med Wochenschr 1997;127:471-8.
19. Cookson B, Johnson AP, Azadian B, Graham Hutchinson JP, Kaufmann M, Woodford N, et al. International inter- and intrahospital patient spread of a multiple antibiotic-resistant strain of *Klebsiella pneumoniae.* J Infect Dis 1995;171:511-3.
